# Towards the Fecal Metabolome Derived from Moderate Red Wine Intake

**DOI:** 10.3390/metabo4041101

**Published:** 2014-12-19

**Authors:** Ana Jiménez-Girón, Irene Muñoz-González, Pedro J. Martín-Álvarez, María Victoria Moreno-Arribas, Begoña Bartolomé

**Affiliations:** Institute of Food Science Research (CIAL), CSIC-UAM, C/Nicolás Cabrera, 9, Campus de Cantoblanco, 28049 Madrid, Spain; E-Mails: irene.munoz@csic.es (I.M.-G.); pedroj.martin.alvarez@csic.es (P.J.M.-A.); victoria.moreno@csic.es (M.V.M.-A.); b.bartolome@csic.es (B.B.)

**Keywords:** wine, polyphenols, fecal metabolome, UPLC-ESI-MS/MS, UHPLC-TOF MS

## Abstract

Dietary polyphenols, including red wine phenolic compounds, are extensively metabolized during their passage through the gastrointestinal tract; and their biological effects at the gut level (*i.e.*, anti-inflammatory activity, microbiota modulation, interaction with cells, among others) seem to be due more to their microbial-derived metabolites rather than to the original forms found in food. In an effort to improve our understanding of the biological effects that phenolic compounds exert at the gut level, this paper summarizes the changes observed in the human fecal metabolome after an intervention study consisting of a daily consumption of 250 mL of wine during four weeks by healthy volunteers (*n =* 33). It assembles data from two analytical approaches: (1) UPLC-ESI-MS/MS analysis of phenolic metabolites in fecal solutions (targeted analysis); and (2) UHPLC-TOF MS analysis of the fecal solutions (non-targeted analysis). Both approaches revealed statistically-significant changes in the concentration of several metabolites as a consequence of the wine intake. Similarity and complementarity between targeted and non-targeted approaches in the analysis of the fecal metabolome are discussed. Both strategies allowed the definition of a complex metabolic profile derived from wine intake. Likewise, the identification of endogenous markers could lead to new hypotheses to unravel the relationship between moderate wine consumption and the metabolic functionality of gut microbiota.

## 1. Introduction

Moderate wine consumption has been associated with preventive effects against cardiovascular diseases, lower incidence of hypertension, diabetes and certain types of cancer [1,2]. Furthermore, a recent intervention study has reported selective modulation of the microbial population by red wine intake that would promote gut health benefits [3]. However, it has become evident that the biological activity and the health effects derived from the consumption of polyphenols-rich foods, such as red wine, are mainly due to the phenolic metabolites formed in the gastrointestinal tract rather than the original forms present in foods [4,5]. Generally, wine polyphenols are poorly absorbed in the small intestine, reaching the colon, where they are catabolized by the gut microbiota, originating a great number of phenolic metabolites that can be further absorbed and also excreted in feces [6].

Human intervention studies are essential to understand the scientific basis of the relationship between food and/or its components and human microbiota. In an integrated way, human intervention studies take into account the inter-individual variability of the human microbiota, as well as the continuous intake of the food and the whole effects on intestinal health. Referring to wine, most of the intervention studies have been conducted with the aim of studying the colonic metabolism of wine polyphenols reflected in urine and plasma [7,8]. In relation to feces, several authors have used *in vitro* models that simulate the conditions of the gastrointestinal tract in order to evaluate the metabolism of polyphenols by gut bacteria and also the impact of the polyphenols and/or their metabolites on the growth and metabolic activity of gut microbiota [9,10,11,12]. To our knowledge, only a pilot study with eight volunteers [13], firstly, and, secondly, a large trial study considering a more relevant number of volunteers (*n =* 41) [14] have been carried out to assess the phenolic metabolite content in feces after wine intake.

Recently, the application of omics technologies have stimulated an increasing interest in food and nutrition science, since they can provide new and important information about the biochemical, molecular and cellular mechanisms that underlie the beneficial or adverse effects of certain bioactive food components [15]. Among omics levels, metabolomics is the one allocated at the end points of the “omics cascade”, because it is the nearest to the phenotype [16]. The Metabolomics Society defines metabolomics as the “newly emerging field of -omics research concerned with the comprehensive characterization of the small molecule metabolites in biological systems which can provide an overview of the metabolic status and global biochemical events associated with a cellular or biological system” [17]. Metabolomics studies will also help us to gain further insight into human metabolic pathways regarding their relationship with diet factors. Mostly, urinary and plasma metabolomes have been studied in different nutritional intervention studies [18,19,20,21,22], including the study of the metabolomic impact of wine intake [8,23]. The influence of the gut microbiome and its interaction with the host is essential to understanding nutrition and metabolism [24]. Therefore, the study of the fecal metabolome may be a potent strategy for understanding interactions between nutrients, the intestinal metabolism and the microbiota composition in health and disease [25]. In relation to the impact of wine intake on the fecal metabolome, only Jacobs *et al.* [26] have attempted ^1^H-NMR metabolite profiling in healthy volunteers who followed a polyphenol-reach diet (grape juice combined with wine extract) over a period of four weeks.

With the final aim of evaluating the potential biological effects of moderate wine consumption on human microbiota and the metabolism involved, this paper compiles the changes observed in the human fecal metabolome after an intervention study involving 41 healthy volunteers. Data were evaluated following two analytical approaches: (1) UHPLC-ESI-MS/MS analysis of phenolic metabolites in fecal solutions (targeted analysis) [14]; and (2) LC-TOF MS analysis of the fecal solutions (non-targeted analysis) [27].

## 2. Experimental Section

### 2.1. Chemicals

All chemicals were of analytical grade. Formic acid was from Riedel-de Haën (Seelze, Germany). Acetic acid was purchased from Scharlau (Barcelona, Spain). Acetonitrile and water were of MS grade and purchased from Labscan (Gliwice, Poland). For the targeted analysis, standards of mandelic acids, benzoic acids, phenols, hippuric acids, phenylacetic acids, phenylpropionic acids and cinnamic acids were purchased from Sigma-Aldrich Chemical Co. (St. Louis, MO, USA), Phytolab (Vestenbergsgreuth, Germany) or Extrasynthèse (Genay, France). The standards 5-(3’,4’,-dihydroxyphenyl)-γ-valerolactone and 5-(4’-hydroxyphenyl)-γ-valerolactone were previously synthesized [28]. The compound 4-hydroxybenzoic 2,3,5,6-d_4_ acid, used as the internal standard (I.S.), was purchased from Sigma-Aldrich Chemical Co. For the non-targeted analysis, a commercial standard mixture containing 42 low molecular weight compounds (acids, bases and neutrals, ABN) was purchased from Sigma-Aldrich (St. Louis, MO, USA) and used to assess instrument variability along the study. Commercial standards of xanthine, glutaric acid, L-lysine, ascorbic acid, pyruvic acid, fumaric acid, butyric acid, isobutyric acid, valeric acid, isovaleric acid, γ-valerolactone, L-ornithine monohydrochloride, 2-methyl amino benzoic acid and methyl 2-aminobenzoate were also purchased from Sigma-Aldrich Chemical Co. (St. Louis, MO, USA) and used for identification purposes in the non-targeted analysis. Stercobilin and urobilinogen were obtained from Santa Cruz Biotechnology, Inc. (Santa Cruz, CA, USA) and also used for identification purposes.

### 2.2. Red Wine

The young red wine (Pinot Noir, vintage 2010), provided by Miguel Torres winery (Spain), was selected for the present study because of its relatively high phenolic content: total polyphenols = 1758 mg of gallic acid equivalents/L, total anthocyanins = 447 mg of malvidin-3-O-glucoside/L and total catechins = 1612 mg of (+)-catechin/L. The antioxidant capacity of the wine measured as ORAC (oxygen radical absorbance capacity) was 35.5 mmol of Trolox equivalents/L. Ethanol, pH, total acidity and volatile acidity were determined in the wine according to the international methods of the OIV (The International Organization of Vine and Wine, 1990). The resulting properties were as follows: the wine pH was 3.52; alcohol degree: 13.8% v/v, total acidity: 6.45 g/L tartaric acid; and volatile acidity: 0.56 g/L acetic acid.

### 2.3. Human Intervention Study Design

A randomized and controlled 4-week intervention study involving 41 healthy volunteers (33 case and 8 control subjects; 22 women and 19 men; age range of 20–65 years) was performed [14]. The participants were not suffering from any disease or intestinal disorder and were not receiving antibiotics or any other medical treatment for at least 6 months before the start of the study or during the study (including the washout period). All of the participants were fully informed about the study and gave written informed consent. The study was carried out according to the rules and approval of the Bioethics committee of Hospital “Ramón y Cajal” (Madrid, Spain).

The study was divided into two consecutive periods: (1) an initial washout period of 2 weeks (baseline) during which the volunteers did not consume any wine or any other alcoholic beverage and followed a low-polyphenols diet; and (2) a period of 4 weeks during which the case volunteers consumed a daily intake of red wine (250 mL) over 28 days (intervention period), divided into two doses. During this latter period, participants also maintained the restrictions for any other alcoholic beverages and followed a low-polyphenols diet. The control group (*n =* 8) followed the same pattern as the intervention group (*n =* 33), with the exception that no wine was ingested during this 4-week period.

Each participant provided samples of feces at two points: (1) after the washout period; and (2) at the end of the study. In total, 66 samples from the intervention group (33 before and 33 after wine consumption) and 16 samples from the control group (8 before-and 8 after the 4-week study period) were collected. Feces were immediately frozen and stored at −80 °C awaiting analysis.

### 2.4. Preparation of Fecal Solutions

Samples were thawed at room temperature and weighed (1.0 g) in 15-mL sterile conical tubes. For the targeted analysis of phenolic metabolites, 10 mL of sterile saline solution (NaCl 0.9%, Fresenius Kabi, Spain) spiked with the I.S. was added, vortexed and centrifuged (10 min, 10,000 rpm, 4 °C) twice. The supernatant (fecal solution) was filtered (0.22 μm) and diluted with acetonitrile (1:4, v/v, acetonitrile/fecal solution). The saline solution contained 3.125 μg/mL of I.S. to achieve a final concentration of 2.5 μg/mL. For the non-targeted analysis, fecal solutions were prepared as previously described and diluted once more with water (1:3, v/v, sample/water) before analysis.

### 2.5. Targeted Analysis of Phenolic Metabolites

Phenolic metabolites were determined in the fecal samples, and data were reported elsewhere [14]. Briefly, phenolic compounds were analyzed by an UHPLC-ESI-MS/MS method validated for the screening of more than 60 microbial-derived phenolic metabolites, including phenols, valerolactones and mandelic, benzoic, hippuric, phenylacetic, phenylpropionic, valeric and cinnamic acids. The chromatographic conditions and MS/MS parameters (cone voltage, collision energy and multiple reaction monitoring, MRM, transition) of the phenolic compounds targeted in our study were previously reported [29].

### 2.6. Non-Targeted Analysis: Metabolomic Study and Metabolite Identification

Metabolomic analysis of the fecal samples was performed using a UHPLC-ESI-TOF MS method previously described [27]. Briefly, internal and external mass calibration of the TOF MS was carried out during the experiments. Two microliters of fecal solution were injected, and each sample was analyzed in duplicate. Quality control (QC) samples containing equal volumes of all of the fecal solutions under study and ABN standards mixture were injected regularly throughout the run to monitor the performance, stability and reproducibility of the UHPLC-TOF MS method, showing a reproducibility of 7.3% and 1.5% inter-day RSD for peaks areas and retention times, respectively.

All raw UHPLC-TOF MS data were extracted and converted to the MS exchange format, mzXML, using the open-source program, Trapper Version 4.3.0 (available at http://tools.proteomecenter.org/wiki/index.php?title=Software:trapper). Data processing was performed using MZmine software, Version 2.7.2 (available at http://mzmine.sourceforge.net/) [30] to obtain a list of peak areas, retention times and accurate mass-to-charge ratios (m/z). Filtering of the data was then carried out to ensure ions with a high quality. Finally, adducts of the same metabolite were grouped, and the resulting output data table of high quality time-aligned detected compounds, with their corresponding retention time, m/z and peak area obtained for each sample, was submitted for statistical analysis.

The assignment of statistically different (*p* < 0.05) metabolites was carried out by matching the obtained accurate m/z to those published in the selected databases, namely, Kyoto Encyclopedia of Genes and Genomes (KEGG) [31], the Human Metabolome Database (HMDB) [32] and the Metabolite and Tandem MS Database (Metlin) [33], within a mass accuracy window of 10 ppm. When available, co-injection of standards with fecal samples was performed to confirm the tentatively identified metabolites. An aliquot of the wine used in the intervention study was also injected in the system to confirm tentative identification of wine compounds detected in the feces.

### 2.7. Statistical Analysis

For both targeted and non-targeted studies, the Shapiro–Wilk test was applied to verify the normal distribution of the data. The comparison of normally distributed data was performed using the two-sample *t*-test with the Welch’s correction (when there were different variances between groups). The non-parametric Mann–Whitney test and the non-parametric Wilcoxon matched-pairs test were used to compare the means of non-normally distributed data. For both studies, a value of *p* = 0.05 was fixed for the level of significance of the mentioned univariate tests. Furthermore, principal component analysis (PCA), from the correlation matrix, was applied to study variability among samples and metabolites and possible trends in the variables due to wine consumption in the non-targeted study. The STATISTICA program for Windows, Version 7.1 (StatSoft Inc., Tulsa, USA, www.statsoft.com), was used for data processing.

## 3. Results and Discussion

**Figure 1:**
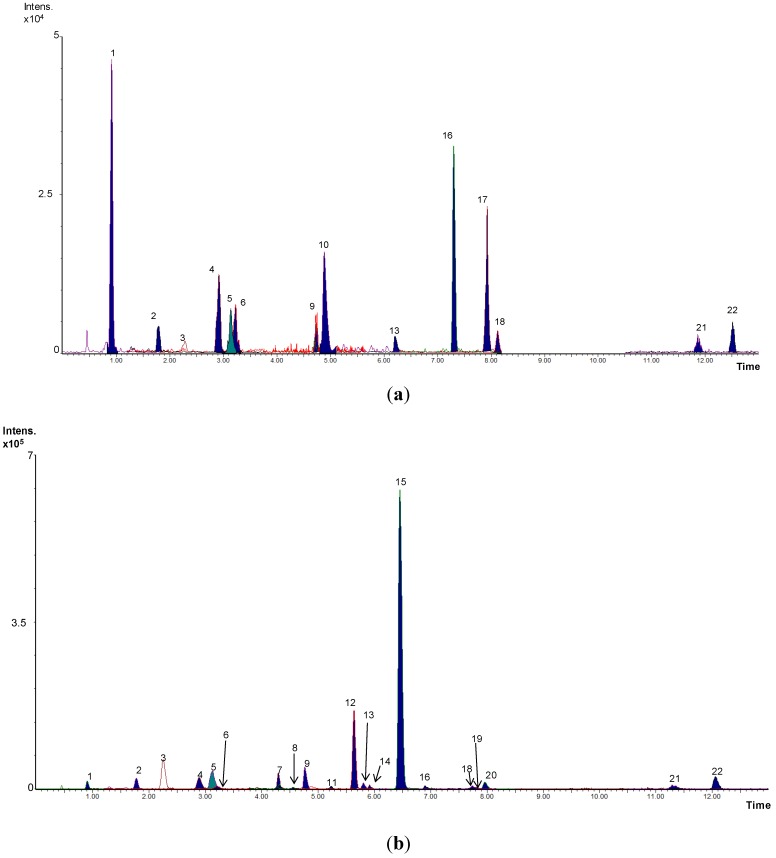
Overlapped MRM chromatograms before (**a**) and after (**b**) wine intake obtained for Volunteer 1. (1) Gallic acid; (2) 3,5-dihydroxybenzoic acid; (3) 3-O-methylgallic acid; (4) 3,4-dihydroxyphenylacetic acid; (5) protocatechuic acid; (6) 4-hydroxybenzoic acid; (7) 4-hydroxy-5-(3’,4’-dihydroxyphenyl)-valeric acid; (8) vanillic acid; (9) 3-hydroxyphenylacetic acid; (10) 3-(4-hydroxyphenyl)-propionic acid; (11) syringic acid; (12) 5-(3’,4’-dihydroxyphenyl)-γ-valerolactone; (13) *p*-coumaric acid; (14) 4-hydroxy-5-(3’-hydroxyphenyl)-valeric acid; (15) 3-(3-hydroxyphenyl)-propionic acid; (16) ferulic acid; (17) benzoic acid; (18) phenylacetic acid; (19) salicylic acid; (20) 5-(3’-hydroxyphenyl)-γ-valerolactone; (21) phenylpropionic acid; and (22) 4-hydroxy-5-(phenyl)-valeric acid.

This study summarizes previous research that examined the impact of moderate wine intake on the human fecal metabolome. In a targeted approach by UPLC-ESI-MS/MS, 35 phenolic metabolites, including mandelic acids, benzoic acids, phenols, hippuric acids, phenylacetic acids, phenylpropionic acids, valeric acids, valerolactones and cinnamic acids, were quantified in the fecal samples, although in a variable number of cases, for both control and intervention groups [14]. As expected, most of the metabolites were found in a higher number of volunteers after wine intake than before the intervention. As an example, Figure 1 shows the MRM chromatograms obtained for Volunteer 1 before and after wine consumption.

No significant differences (*p* > 0.05) were found in the basal (before wine consumption) content of phenolic metabolites between the control and intervention groups (from the Mann−Whitney test); the total phenolic metabolite basal content being 175 ± 98 and 358 ± 270 μg/g feces for the control and intervention groups, respectively. The content of phenolic compounds at baseline obtained in our targeted study [14] (data not shown) was included within the concentration ranges previously reported in the literature, in spite of the differences in methodology, number of volunteers and sample preparation across studies.

**Figure 2:**
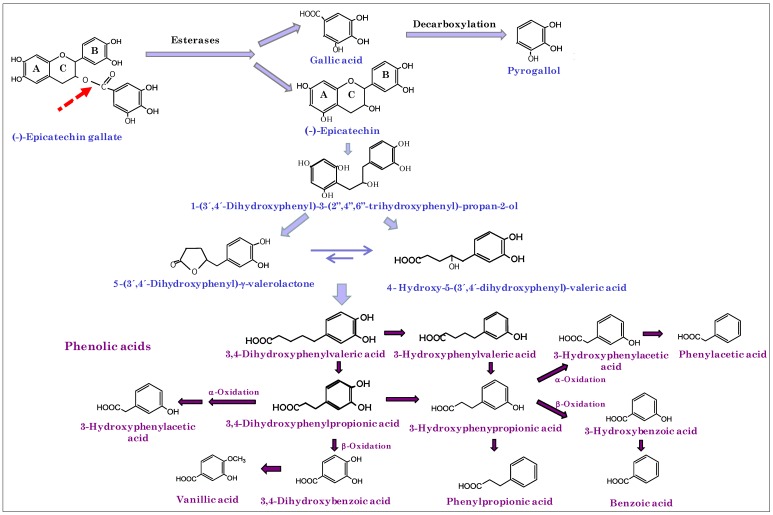
Metabolic pathway for the degradation of monomeric flavans-3-ol by gut microbiota.

**Figure 3:**
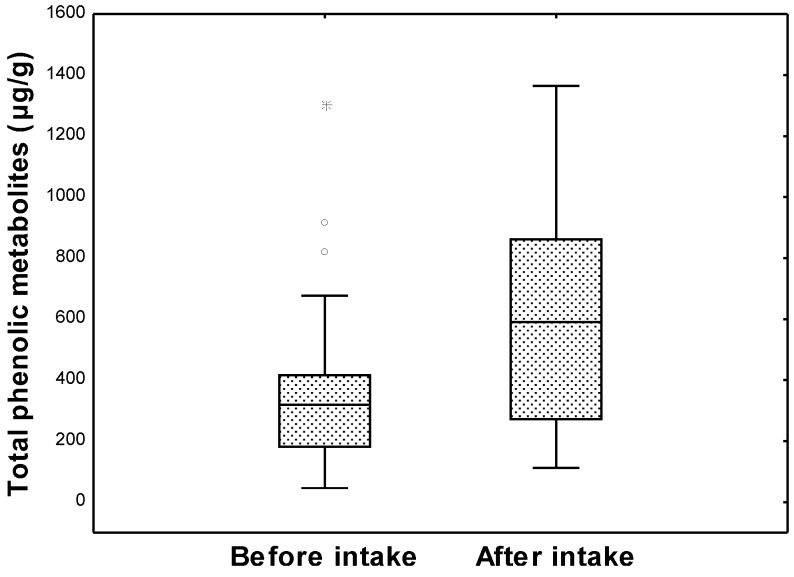
Box and whiskers plots (^_^: median; 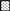: 25th–75th percentiles; 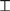: non-outlier range; ○: outliers; 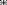: extremes) of the total phenolic metabolites content in the intervention group (*n =* 33).

Figure 2 represents the metabolic pathway for the degradation of monomeric flavan-3-ols by gut microbiota giving rise to the detected derivatives. Oligomers and polymers of flavan-3-ols are the major wine phenolic compounds that reached the colon intact. The catabolism of dimeric procyanidins involves C-ring opening, followed by lactonization, decarboxylation, dehydroxylation and oxidation reactions, among others [6]. The initial microbial metabolism of wine flavan-3-ols, such as (-)-epicatechin and procyanidin, produces 1-(3’,4’-dihydroxyphenyl)-3-(2”,4”,6”-trihydroxyphenyl)-propan-2-ol, which subsequently is converted into 5-(3’,4’-dihydroxyphenyl)-γ-valerolactone and 4-hydroxy-5-(3’,4’-dihydroxyphenyl)-valeric acid [6,10,29] (Figure 2). Shortening of the side-chain length of valeric acids by subsequent β-oxidation reaction resulted in phenylpropionic, phenylacetic and benzoic acids [6]. It is important to mention, that some of these compounds, such as benzoic, phenylacetic, 3-phenylpropionic and 4-hydroxy-5-phenylvaleric acids, were the most abundant phenolic metabolites found at baseline (before wine consumption) by the UPLC-ESI-MS/MS approach (targeted approach). For the control group, no significant differences (*p* > 0.05) between samples before and after the four-week study period were found (from the non-parametric Wilcoxon matched-pairs test), as expected. Nevertheless, for the intervention group, after applying Wilcoxon test, significant differences (*p* < 0.05) between samples before and after moderate wine consumption were found for the content of 10 phenolic metabolites (Table 1), which mainly come from the catabolism of both flavan-3-ols and anthocyanins, the major flavonoids in red wine. Furthermore, the total phenolic metabolites content was significantly higher (*p* < 0.05) in the samples after wine intake (625 ± 380 µg/g feces) in comparison to the samples before intake (358 ± 270 µg/g feces). To represent these data, Figure 3 displays the box and whiskers plots (median, 25th and 75th percentiles, non-outlier range, outliers and extremes) of the sum of the contents of targeted phenolic metabolites before and after red wine intervention. In general, the 25th–75th percentiles were greater for the samples after wine intake, indicating that wine polyphenol metabolism enhances inter-individual variability further still. No detectable amounts of the phenolic compounds present in the wine (anthocyanins, flavan-3-ols, flavonols or stilbenes) were found in the fecal samples, indicating that they were completely metabolized during their passage through the gastrointestinal tract.

**Table 1:** Fecal metabolites significantly regulated by moderate wine consumption.

Compound	Analysis		Effect of Wine Intake (↑, ↓)
	Targeted	Non-Targeted	
**Group I: Wine compounds**			
2-Hydroxyglutaric acid ^a^		+	↑
2-Methylbutyric acid		+	↑
2,3-Pentanedione		+	↑
Diethylmalonate		+	↑
2-Phenethyl butyrate		+	↑
2-Phenylethyl hexanoate		+	↑
**Group II: Phenolic metabolites**			
*Benzoic acids*			
3,5-Dihydroxybenzoic acid	+		↑
Protocatechuic acid	+		↑
3-*O*-Methylgallic acid	+		↑
Vanillic acid	+		↑
Syringic acid	+		↑
Benzoic acid ^b^		+	↑
*Phenylacetic acids*			
3-Hydroxyphenylacetic acid	+		↑
*Phenylpropionic acids*			
3-Phenylpropionic acid	+		↑
3-(3’-Hydroxyphenyl) propionic acid ^b^		+	↓
*Valeric acids*			
4-Hydroxy-5-(3’-hydroxyphenyl) valeric acid		+	↑
4-Hydroxy-5-(phenyl) valeric acid	+	+	↑
4-Hydroxy-5-(3’,4’-dihydroxyphenyl) valeric acid	+		↑
*Valerolactones*			
5-(3’,4’-Dihydroxyphenyl)- γ-valerolactone		+	↑
5-(3’-Hydroxyphenyl)-γ-valerolactone	+		↑
**Group III: Endogenous metabolites**			
Xanthine ^b^		+	↓
Tricarballylic acid		+	↓
Glutaric acid ^b^		+	↑
Urobilinogen ^b^		+	↑
Stercobilin ^b^		+	↑
Docosahexaenoic acid methyl ester		+	↑
Deoxycholic acid		+	↑
Sulfolithocholic acid		+	↓
Cholesterol sulfate		+	↑

^a^ Confirmed in the wine used in the study; ^b^ metabolite identification confirmed with commercial standards in the non-targeted approach.

In line with this, other authors have reported significant increases in the urinary concentration of phenolic metabolites (both phase II and microbial-derived phenolic metabolites) after wine interventions [34]. In particular, the highest increase in urine after wine consumption was found for microbial metabolites derived from anthocyanins, such as syringic, p-coumaric, gallic acids and pyrogallol, and from flavan-3-ols, such as hydroxyphenylvalerolactones. These results are consistent with the significant increases obtained in our targeted study on feces after wine consumption [14]. Moreover, among the hydroxybenzoic acids found in urine, the highest increase was observed for syringic acid (2.78-fold increase after wine consumption), while the other hydroxybenzoic acid metabolites exhibited increases ranging from 1.33- to 1.93-fold. This is in accordance with our targeted study on feces; the highest increase was also found for syringic acid (2.4-fold increase after wine consumption), while the other hydroxybenzoic acid metabolites exhibited increases ranging from 1.04- to 1.67-fold. Nevertheless, no significant differences were observed for protocatechuic acid in urine after wine consumption, while we found a significant increase in feces (1.67-fold). Regarding phenols, the highest increase in urine was found for 3-hydroxyphenylacetic acid (2.29-fold), which also showed the highest increase in our study on feces (2.05-fold). In relation to plasma samples, Cacceta *et al.* [35] observed significant increases in 4-*O*-methylgallic acid and caffeic acid after wine consumption, while no significant differences were observed for protocatechuic acid. On the contrary, in our targeted analysis [14], we did not observe a significant increase of the two first metabolites in feces, while, as mentioned above, a significant increase was found for protocatechuic acid in feces after wine consumption.

Finally, an attempt to distribute the volunteers by means of the total phenolic metabolite content in fecal solutions was carried out. For the control group (*n =* 8), the distribution of the volunteers was similar before and after the four-week intervention period. For both times, the maximum frequency (four volunteers) was observed for the interval 100–150 µg/g feces of total phenolic metabolite content. Interestingly, the frequency histogram for the intervention group (*n =* 33) became wider and moved towards higher values after the four-week wine intake [14]. Thus, three groups could be established according to the total phenolic metabolites content in feces after wine intervention: (1) low metabolizers: <500 µg/g feces; (2) moderate metabolizers: 500–1000 µg/g feces; and (3) high metabolizers: >1,000 µg/g feces [14]. The group with total phenolic metabolite content <500 µg/g feces included 27 volunteers before the wine intake, but only 13 after the intake. In contrast, the number of volunteers in the group exhibiting a total phenolic content of 500–1,000 µg/g feces increased from five to 13 volunteers after the wine intake. The same was observed for the group of highest phenolic metabolite content (>1,000 µg/g feces), the number of volunteers increased from one to seven after the wine intake. In line with this, other authors [36,37] have previously suggested a particular distribution of the volunteers according to their phenolic-metabolizing capacity after an intervention study with phenolic-rich foods. These results suggest that a different gut microbial capacity to metabolize wine polyphenols exists among the human population, as observed for polyphenols from other sources.

For the non-targeted approach, an UHPLC-TOF MS method was applied [27]. The overall metabolic differences between intervention and control groups and differences obtained before and after wine consumption, in the intervention group, were first evaluated by PCA. Similarly to the targeted study results, for the control group, no separation was observed between samples before and after the four-week study period. Nevertheless, when the wine consumers group was considered, fecal samples before and after wine intake were not discriminated neither, suggesting that the vast majority of the metabolome was not altered due to wine consumption, as expected in a non-targeted metabolomic approach. Taking advantage of the multilevel structure in a crossover designed metabolomics study, other authors have suggested the use of multilevel Partial Least Squares-Discriminant Analysis (PLS-DA) as a possible alternative to detect the significant metabolites after intervention [38]. In the present work, the differences in the metabolomic profile of the wine consumers group were evaluated using the non-parametric Wilcoxon matched-pairs test. We found that 70 metabolites showed significant differences (*p* < 0.05) before and after wine consumption. These 70 significantly different metabolites were checked in the control group, and those found differentially expressed also in the control group (*i.e.*, not associated with wine intake) were removed from the analysis. Finally, the MS responses of 37 metabolites were found to be significantly different before and after wine consumption. The tentative identification of the 37 metabolic differences related to moderate wine consumption was attempted by searching in metabolomics databases and the literature, and when available, commercial standards were used to confirm or discard initial metabolite identification (Figure 3). After an exhaustive investigation, a total of 20 metabolites were tentatively or completely identified (Table 1), whereas the rest of them (17 compounds) remained unknown (data not shown) and were not discussed. The tentatively or completely identified metabolites were classified into three groups (I–III). Group I corresponded to compounds that had been previously reported in wine (*n =* 6). Group II comprised phenolic metabolites derived from the action of gut microbiota on wine polyphenols (*n =* 5), and finally, Group III included endogenous metabolites and other compounds derived from non-phenolic nutrient pathways (*n* = 9) (Table 1).

As expected, the content of all wine compounds (Group I) significantly increased after wine intake (Table 1). The presence of all of these in wine has been reported in the literature [27]. Moreover, 2-hydroxyglutaric acid was detected in the same wine used in this study. Finally, 2-methylbutyric acid has been reported also in feces [39]. These results confirmed the intact passage of certain wine compounds through the gastrointestinal tract.

Microbial-derived metabolites (phenolic metabolites) (Group II) constituted the most abundant group of compounds that showed statistically-significant differences in their MS response after wine intake. As expected, some differences were observed between both studies. In the non-targeted analysis, benzoic acid, 3-(3’-hydroxyphenyl) propionic acid, 4-hydroxy-5-(3’-hydroxyphenyl) valeric acid, 4-hydroxy-5-(phenyl) valeric acid and 5-(3’,4’-hihydroxyphenyl)-γ-valerolactone were found to be significantly regulated after wine intake (Table 1). All of these compounds were also identified in the targeted analysis [14], although significant differences in their MS response after wine intake were only found for 4-hydroxy-5-(phenyl) valeric acid. In particular, the targeted analysis led to a higher number of significant phenolic metabolites (*n =* 10). These differences were attributed to differences in sensitivity between both methodologies. In both studies, phenolic metabolites were found upregulated by wine intake, except 3-(3-hydroxyphenyl) propionic acid (Table 1), a microbial metabolite also derived from the catabolism of flavan-3-ols. This may be due to a high dehydroxylation activity of the intestinal microbiota resulting in phenylpropionic acid, as previously reported with grape seed polyphenols [40]. The biological relevance of the dehydroxylation activity of the intestinal microbiota could be related to the antimicrobial effect of the produced metabolites, among others [6]. Cueva *et al.* [41] stated that the number and position of substitutions in the phenolic acids’ benzene ring influenced their antimicrobial potential against lactobacilli, but in a different manner from that in the *E. coli* species. As an example, for phenylpropionic acids, growth inhibition of *E. coli* strains was as follows: non-substituted > 4-hydroxy- > 3-hydroxy- > 3,4-dihydroxy-substituted acid.

**Figure 4:**
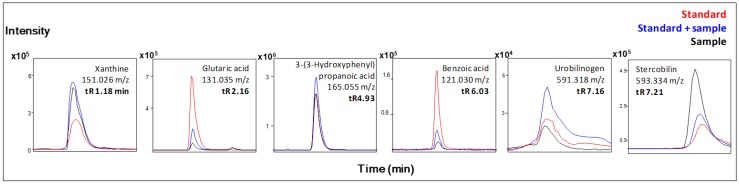
Extracted ion chromatograms of a fecal sample (black line), fecal sample spiked with 2.5 µg/mL 3-(3’-hydroxyphenyl) propionic acid, 1 µg/mL glutaric acid, 1 µg/mL benzoic acid, 1 µg/mL xanthine, 3.3 µg/mL stercobilin and 1.2 mg/mL urobilinogen (blue line) and commercial standard (red line).

Finally, among metabolites included in Group III (endogenous metabolites and other compounds derived from non-phenolic nutrient pathways), xanthine, glutaric acid, urobilinogen and stercobilin were confirmed co-injecting the commercial standards (Figure 4). Xanthine is an intermediate in the degradation of adenosine monophosphate to uric acid, being formed mainly by oxidation of hypoxanthine by xanthine oxidase. We found a lower concentration of xanthine in feces after wine intake. This is in line with previous studies that suggest that the antioxidant properties assigned to flavonoids [42] and resveratrol [43,44] are due to their inhibitory effect on the activity of xanthine oxidase. Glutaric acid has been previously reported on feces [39]. It is a dicarboxylic acid produced during the metabolism of some amino acids, and also, it can be produced by anaerobic fermentative gut bacteria (together with lactic acid) [45]. In our non-targeted analysis, we found a higher content of glutaric acid in feces after wine intake. Finally, urobilinogen and stercobilin, byproducts of bilirubin degradation, were found to be regulated by wine intake. Urobilinogen is formed in the intestines by bacterial action, especially *Clostridium* spp. [46]. Part of it is reabsorbed, taken up by the hepatocytes into the circulation and excreted by the kidney. The urobilinogen remaining in the intestine is oxidized to brown stercobilin. In our study, the content of both metabolites significantly decreased after wine intake, which is in accordance with numerous studies that indicate that flavan-3-ol-rich sources, such as wine, may modulate the intestinal microbiota, increasing beneficial bacteria, but inhibiting other groups, such as *Clostridium* spp. [47,48,49,50,51,52].

As mentioned above, volunteers were classified into three groups by looking at their capacity to metabolize wine polyphenols. In order to assess if the metabolizer capacity of the volunteers had an influence on the metabolomic profile after moderate wine consumption, the Wilcoxon matched pairs test was applied separately to the low, moderate and high metabolizers groups. When low metabolizers were evaluated, similar results were obtained for all metabolites, except for sulfolithocholic acid, which was found not to be regulated by wine consumption. For the moderate metabolizers group, also similar results were obtained (*p* < 0.10), except for 4-hydroxy-5-(3’-hydroxyphenyl) valeric acid and stercobilin, which were found not to be regulated by wine intake. Interestingly, the main differences were found when the high metabolizers group, with only seven individuals, was evaluated separately. In this group, the content of benzoic acid, 4-hydroxy-5-phenylvaleric acid, urobilinogen, docosahexaenoic acid methyl ester, deoxycholic acid, sulfolithocholic acid and cholesterol sulfate were not found significantly different after wine intake. Since these metabolites are either derived or regulated by gut microbiota, we hypothesize that microbial-derived metabolites of wine polyphenols may be related to changes in the microbiota functionality. Nevertheless, more studies regarding the characterization and metabolic activity of the fecal microbiota from these samples need to be carried out to confirm this assumption.

## 4. Conclusions

In conclusion, targeted and non-targeted approaches have been used to study the impact of moderate wine intake on the fecal metabolome of healthy volunteers. The targeted study has proven that moderate wine intake significantly promotes changes in the profile and content of phenolic metabolites derived from the action of gut microbiota on wine polyphenols. Moreover, it has been observed that wine polyphenol metabolism enhanced inter-individual variability. Complementary to this, the results derived from the non-targeted approach showed that phenolic metabolites seemed to profoundly impact the global fecal metabolite profile. Furthermore, the results derived from the non-targeted approach have extended the list of components regulated by wine intake. In particular, the intact passage of certain wine compounds through the gastrointestinal track (*i.e.*, 2-hydroxyglutaric acid) was confirmed. In addition, it was observed that wine consumption impacts other nutrient pathways (*i.e.*, lipids) and endogenous metabolites (*i.e.*, xanthine and bilirubin). Overall, the differences observed in the fecal metabolome after wine consumption may reflect changes in microbiota composition and/or functionality that could, in turn, be partly attributed to modulation effects by wine polyphenols and/or their metabolites. Future work will focus on the metagenomic analysis of these fecal samples, which will probably confirm the hypothesis that we are suggesting in this paper.
